# The Institutional Treatment of Consumption

**Published:** 1916-05-06

**Authors:** 


### THE INSTITUTIONAL TREATMENT OF CONSUMPTION.

### I.—The Sorting of the Patients.

Provision for the treatment of consumption in institutions will necessarily loom largely in any scheme for combating this disease, though it is a fair assumption that dispensaries will in the future increase both in importance and in number. With the return of something approaching normal conditions greater supervision and control of the tubercular population may be expected to radiate from these centres, and in preventive work they will become factors of the highest importance.

### The Selection of Sanatorium Cases.

In considering the institutional treatment of consumptives it is now customary to differentiate more and more strictly between sanatorium treatment and the treatment of hospital cases. The former connotes the attempt to produce definite arrest of the disease in certain amenable types or, at least, to bring the lesions to a state of quiescence likely to be of some duration under reasonably favourable conditions, and this with a fairly complete restoration of the general health. The "hospital" cases should be (1) acutely ill cases of apparently recent origin; (2) those who are worth patching up, but who are unlikely to have their disease arrested for any considerable length of time; (3) patients whom it is desirable to teach how to live so as to be in the least degree dangerous to others; and (4) those who can be persuaded, in default of compulsory powers, to submit to segregation for prophylactic reasons and for the benefit of the community, even if only temporarily. There remains still the hopelessly advanced case who is, under present conditions, too often discharged to his home to die, or transferred thence on the urgent insistence of the relatives. Such cases, by reason of their very helplessness, are a source of great danger in their homes, and require much more supervision than can be given by a visiting nurse or by the best-intentioned relatives. More institutional provision is needed for these unfortunate people, but as this would be in the nature of a home for the dying, it would be manifestly unfair and unwholesome to reserve a ward in a hospital block for such cases. Even provision in a separate block in the same grounds cannot be recommended; the depressing news of deaths is sure to get around. Totally separate institutions are required, and as they will not be used for the purpose of treatment they will have to be paid for out of public health funds or supported by voluntary contributions, and not by money derived from sanatorium benefit funds.

### The Problem of the Moribund Case.

Under working conditions there will always be a tendency for institutions, or parts of institutions, reserved for "hospital" cases to acquire an evil reputation among patients of a given area. Even if hopeless cases nearing their end are—as is rarely the case—provided for elsewhere, or sent home, a certain number of deaths will occur in these wards, and consequently the offer of a bed therein is apt to be looked on as equivalent to an unfavourable verdict. An attempt to overcome this difficulty has been made in some places by sending to hospitals persons in whom the diagnosis is not clear, and whom it is wished to observe or to submit to tests before coming to a conclusion. These persons are, of course, not in the least suggestive of moribund patients, and they may therefore help to dispel the above fatalistic conception. But, on the other hand, it must not be forgotten that these "observation cases" run a certain risk by being mixed up with "open" cases; this risk in a well-conducted institution is admittedly small. The plan savours of subterfuge, and its object is not likely long to be successfully achieved.

### The Interpretation of Sanatorium Results.

The fact that not a few murmurings have appeared of late in the provincial Press against the alleged poor results of "sanatorium" treatment shows that the general public do not yet appreciate the need for stricter differentiation of cases. Sanatorium medical officers constantly have unpromising clinical material thrust upon them, and their results are unfairly distorted. An ideal selection of cases would imply theoretically a discharge list containing on nearly every line the result, "Much improved," and for the rest the term "Improved." Under the former heading is included, for the moment, a result which is even one better—namely, "Disease arrested." This is generally accepted as including absence of T.B. in the sputum, in addition to general restoration of health. "Much improved" also includes this last, but T.B. may still be present, though the lesion is considered "quiescent" as regards physical signs, whether reduced in extent or not. "Improved" may be taken as meaning much the same as regards the local disease and signs, but with the general health not quite fully restored. Such a statement of results is, however, imperfect, as the original state is not taken into account; the patient may have started as ambulant afebrile or with high fever even at rest, and there are intervening grades. Sufficient, however, has been said to indicate how desirable it is that cases of consumption should be most carefully sorted if sanatorium treatment is not to fall into disrepute, and if large sums of money are not to be wasted in merely increasing the expectation of life of certain types, who, under the present system of voluntary discharge, add to the risk to the normal population of contracting tuberculosis.

There is yet another class of patient which remains to be considered. This class is in any scheme for the prevention of tuberculosis the most important of all. We refer to the children. The incidence of pulmonary tuberculosis in children—*i.e.* those below the insurable age of sixteen—does not constitute a very difficult problem. It is with the children in "delicate" health or with enlarged glands who are chiefly discovered in the course of school inspection that the main problem lies. From these children are developed, there can be no doubt, a large proportion of the adult cases of consumption. Provision for what may be designated the prophylactic treatment of children is one of those measures in the fight against tuberculosis of the value of which there are no two opinions. If these children can be taken away from their environment and placed under good hygienic conditions with plenty of good food, there can be no doubt that in many cases the seeds of consumption are prevented from developing, and in every case the child's resistance to other infections is definitely increased. Whether the selected children be diagnosed as definitely tubercular or as merely suffering from retarded physical development, they can hardly fail to gain permanent benefit, and thus the money expended will be in nearly every instance well spent and will yield a satisfactory, if deferred, return to the community. The particular form of institutional treatment designed for these children will partake of the character of an open-air school. In America they use the name "Preventorium." But for the present this aspect of institutional treatment of consumptives must be left for further consideration later.

